# Metabolic Profile in Early Pregnancy Is Associated with Offspring Adiposity at 4 Years of Age: The Rhea Pregnancy Cohort Crete, Greece

**DOI:** 10.1371/journal.pone.0126327

**Published:** 2015-05-13

**Authors:** Vasiliki Daraki, Vaggelis Georgiou, Stathis Papavasiliou, Georgia Chalkiadaki, Marianna Karahaliou, Stella Koinaki, Katerina Sarri, Maria Vassilaki, Manolis Kogevinas, Leda Chatzi

**Affiliations:** 1 Department of Social Medicine, Faculty of Medicine, University of Crete, Heraklion, Greece; 2 Clinic of Endocrinology, University Hospital of Crete, Heraklion, Greece; 3 Centre for Research in Environmental Epidemiology (CREAL), Barcelona, Spain; 4 IMIM (Hospital del Mar Research Institute), Barcelona, Spain; 5 CIBER Epidemiologia y Salud Pública (CIBERESP), Barcelona, Spain; 6 National School of Public Health, Athens, Greece; Karolinska Institutet, SWEDEN

## Abstract

**Context:**

Maternal pre-pregnancy obesity may increase the risk of childhood obesity but it is unknown whether other metabolic factors in early pregnancy such as lipid profile and hypertension are associated with offspring cardiometabolic traits.

**Objective:**

Our objective was to investigate whether fasting lipid, glucose, and insulin levels during early pregnancy and maternal pre-pregnancy weight status, are associated with offspring adiposity measures, lipid levels and blood pressure at preschool age.

**Design and Methods:**

The study included 618 mother-child pairs of the pregnancy cohort “Rhea” study in Crete, Greece. Pregnant women were recruited at the first prenatal visit (mean: 12weeks, SD: 0.7). A subset of 348 women provided fasting serum samples for glucose and lipid measurements. Outcomes measures were body mass index, abdominal circumference, sum of skinfold thickness, and blood pressure measurements at 4 years of age. A subsample of 525 children provided non-fasting blood samples for lipid measurements.

**Results:**

Pre-pregnancy overweight/obesity was associated with greater risk of offspring overweight/obesity (RR: 1.83, 95%CI: 1.19, 2.81), central adiposity (RR: 1.97, 95%CI: 1.11, 3.49), and greater fat mass by 5.10mm (95%CI: 2.49, 7.71) at 4 years of age. These associations were more pronounced in girls. An increase of 40mg/dl in fasting serum cholesterol levels in early pregnancy was associated with greater skinfold thickness by 3.30mm (95%CI: 1.41, 5.20) at 4 years of age after adjusting for pre-pregnancy BMI and several other confounders. An increase of 10mmHg in diastolic blood pressure in early pregnancy was associated with increased risk of offspring overweight/obesity (RR: 1.22, 95%CI: 1.03, 1.45), and greater skinfold thickness by 1.71mm (95% CI: 0.57, 2.86) at 4 years of age.

**Conclusions:**

Metabolic dysregulation in early pregnancy may increase the risk of obesity at preschool age.

## Introduction

Childhood obesity is one of the greatest public health challenges worldwide and is having a major impact on human morbidity, mortality and quality of life [1, 2]. In Europe, its prevalence has increased dramatically in last decades, while recent estimates report that Greece has the highest prevalence of childhood obesity [3]. The commonly held causes of obesity, which are over-eating, inactivity, and genetic pre-disposition, do not fully explain the current obesity epidemic [4]. According to the developmental origins of health and disease (DOHaD) hypothesis changes in the intrauterine environment at critical or sensitive periods of the developmental process could have irreversible, lifelong consequences in offspring metabolism [4, 5]. Metabolic disorders during pregnancy like obesity, gestational diabetes, and excess gestational weight gain are well known exposures that predispose offspring to obesity [6–9]. However, the role of maternal metabolism in the first trimester of pregnancy, which is a critical developmental time window for gestational programming, is unclear [10].

Epidemiological studies indicate that higher maternal pre-pregnancy body mass index is associated with increased risk of childhood obesity [7, 8]. Few studies have examined so far its association with other cardiometabolic risk factors such as lipid levels and blood pressure in children with controversial results [11–15]. Whether these associations reflect direct intrauterine causal mechanisms or are driven in a gender-related manner remains unclear. Animal studies suggest that sex-specific vulnerabilities to an altered *in utero* metabolic environment may mediate sex differences in fetal growth and predisposition to adult diseases, such as cardiovascular disease [16, 17]), however evidence from human studies is scarce [18]. Moreover, it has been suggested that maternal gestational weight gain and smoking during pregnancy can act as confounders of such associations [6, 19, 20], although it can be argued that they may also act as mediators.

Studies on other maternal cardiovascular risk factors such as dyslipidemia or hypertension in pregnancy in association with offspring cardiometabolic health are scarce with conflicting results [21, 22]. Maternal non fasting lipid levels in early pregnancy were shown to be associated with increased offspring’s fat percentage and waist-to-height ratio values at preschool age [22]. Hypertension in early pregnancy has been associated with increased risk of fetal growth restriction [23, 24]), and preeclampsia [23], but there are no studies examining blood pressure in early pregnancy with offspring cardiometabolic traits.

In this study, we aimed to fill these research gaps by investigating the impact of maternal metabolic profile in early pregnancy characterized by pre-pregnancy Body Mass Index (BMI), blood pressure levels, and fasting lipids, insulin and glucose levels on offspring cardiometabolic traits in early childhood, in a prospective pregnancy cohort in Crete, Greece, after controlling for several confounding and mediator factors.

## Materials and Methods

### Study design and population: Rhea cohort

The present study is part of the “Rhea” project, a pregnancy cohort which examines prospectively a population-based cohort of pregnant women and their children at the prefecture of Heraklion, Crete, Greece [25]. We recruited pregnant women (Greek and immigrants) at the time of the first comprehensive ultrasound examination, around week 12 of gestation (mean: 12.1 weeks, SD: 0.7), from four prenatal clinics (two public and two private) in Heraklion city, during the twelve-month period from February 2007 until February 2008. The inclusion criteria for study participants were: residents in the study area; pregnant women aged > 16 years; 1st prenatal visit: hospitals or private clinics at Heraklion district; no communication handicap. The study was approved by the Ethical Committee of the University Hospital of Heraklion (Crete, Greece), and all participants provided written informed consent after complete description of the study.

Of 1363 singleton live births in the Rhea study, 879 children participated at the 4 years follow up, during which anthropometry and non-fasting blood samples were obtained from 785 children. From those, complete data for maternal anthropometry, follow-up interview and child anthropometric measurements were available for 631 mother–child pairs. We excluded women who had been diagnosed with preeclampsia [n = 13 (8 in this, and 5 in previous pregnancies)], since this condition is associated with a higher blood pressure and BMI in childhood and early adult life [21]. Thus, a cohort of 618 mother–child pairs was available for the present analysis. Of them a subset of 348 women provided fasting blood samples for glucose and lipid measurements, due to the timing of enrollment in the study. A subsample of 525 children provided non-fasting blood samples at the 4 year follow up (mean: 4.2 years, SD: 0.2).

### Exposures: maternal pre-pregnancy BMI and metabolic profile during early pregnancy

#### Maternal overweight/obesity

Maternal height, measured at the first prenatal visit, and pre-pregnancy weight, as reported at the first major ultrasound visit, were used to calculate the pre-pregnant body mass index (BMI; weight (kg)/height (m)^2^). Women were divided into 3 categories as follows: no excess weight (pre-pregnant BMI<25 kg/m^2^), overweight (pre-pregnant BMI: 25–29.9 kg/m^2^) and obese (pre-pregnant BMI ≥30kg/m^2^) according to the definitions of the World Health Organization.

#### Maternal fasting glucose and lipid levels in early pregnancy

We measured lipids [total cholesterol (TC), triglycerides (TG), high-density lipoprotein cholesterol (HDL-C)] and glucose by standard enzymatic methods (Medicon, Greece) on an automatic analyzer (AU5400 high-volume chemistry analyzer; Olympus America, Inc., Melville, New York). Low density lipoprotein cholesterol (LDL-C) concentration was estimated by using the formula: LDL-C = TC-[(TG/5) + HDL-C]. C-reactive protein levels were measured with a high-sensitivity homogenous immunoassay (ORS 6199, Beckman Coulter, USA) on an automatic analyzer (AU5400 high-volume chemistry analyzer; Olympus America, Inc., Melville, New York). Maternal insulin concentration was measured by IMMULITE 2000 immunoassay system (Siemens Healthcare Diagnostics, Inc., Deerfield, Illinois). The inter- and intra-assay coefficients of variation were less than 5%.

Maternal abnormal lipid profile in early pregnancy was defined as triglyceride levels ≥150 mg/dL, or total cholesterol levels ≥200 mg/dL, or high density lipoprotein cholesterol (HDL-C) levels <50 mg/dL, or low density lipoprotein cholesterol (LDL-C) levels ≥130 mg/dl [26]. Maternal hyperglycemia in early pregnancy was defined as a maternal fasting blood glucose level ≥92 mg/dl [27].

#### Maternal blood pressure in early pregnancy

Systolic (SBP) and diastolic (DBP) blood pressure were measured at the ultrasound examination. Measurements were taken by using an electronic blood pressure monitor after 10 minutes of rest in a sitting position. All readings were replicated 3 times in the right arm for each woman. The mean value obtained from the second and third readings was used in the analysis [25].

### Outcomes: Offspring cardiometabolic traits during early childhood

#### Child adiposity outcomes at 4 years of age

Child anthropometric measures at 4 years of age (mean: 4.2 years, SD: 0.2), were taken by specially trained research assistants according to standard procedures at the University Hospital of Heraklion, Crete, Greece. Body weight was measured once by a digital scale (Seca Bellisima 841) to the nearest 0.1kg with subjects standing without shoes and in light clothing. Height was measured to the nearest 0.1 cm with the use of a commercial stadiometer (Seca 213). Overweight/obesity were defined using age-and sex-specific BMI thresholds proposed by the International Obesity Task Force [28].

Waist circumference (WC) was measured in duplicate to the nearest 0.1 cm in the standing position, at the high point of the iliac crest at the end of a gentle expiration, using a flexible tape measure (Seca 201). We used age-and sex-specific 90^th^ waist circumference percentiles based on national references [29], as a cut-off point to define central adiposity.

Skin fold thickness was measured to the nearest 0.1mm at four anatomical sites (triceps, thigh, subscapular and suprailiac) on the right side of the body, using calibrated calipers (Harpenden HSK- BI, CE-0120).

#### Child non fasting lipid profile at 4 years of age

We measured non fasting total cholesterol (TC) and high-density lipoprotein cholesterol (HDL-C) with the same biochemical methods used for maternal lipids measurements. As there is no standard definition for lipid disorders at preschool age, we used the 75th percentile of the study cohort distribution for total cholesterol (≥173.9 mg/dL), LDL-C- (≥111.5 mg/dL) and the 25^th^ percentile for HDL-C levels (<40 mg/dL) as a cut-off point to denote abnormal lipid levels in children [30].

#### Child blood pressure at 4 years of age

At 4 years of age, trained research assistants measured systolic (SBP) and diastolic blood pressure (DBP) after 5 minutes rest in the seated position, at the child right arm with a cuff of appropriate size for arm circumference using a Dinamap Pro Care 400, which utilizes an oscillometric method. We used the average of five consecutive measurements, taken with 1 minute intervals [31]. We then calculated blood pressure percentiles specific for age, sex, and height, as blood pressure measurements in children are suggested to differ according to these characteristics.

### Potential confounders

Potential confounders included characteristics that have an established or potential association with maternal metabolic profile in early pregnancy or cardiometabolic risk in childhood including: maternal age at delivery (years); maternal education (low level: ≤6 yrs. of school; medium level: ≤12 yrs. of school; high level: university or technical college degree); maternal origin (Greek/other); marital status (married/other); physical activity before pregnancy (yes/no); parity (primiparous/multiparous); type of delivery (vaginal/caesarean); smoking during pregnancy (yes/no); gestational weight gain, categorized according to 2009 Institute of Medicine guidelines [32]; family history of dyslipidemia (yes/no); family history of diabetes (yes/no); gestational diabetes (yes/no); gestational hypertension (yes/no); gestational age (weeks); birth weight (kg); child’s sex (male/female); duration of breastfeeding (months); day of care attendance at the first 2 years of life (yes/no); TV viewing at 4 years of age (hours/day); child’s energy intake (Kcals/day) at 4 years of age based on a validated food frequency questionnaire [33].

### Statistical analysis

Differences in distributions of normally distributed variables were tested with t-test, non-normally distributed continuous variables were tested by using non parametric tests (i.e., Mann-Whitney, Kruskal-Wallis, and Spearman non parametric statistical tests), whereas categorical variables were tested with chi-square test (Pearson’s or Fisher exact test with Monte-Carlo correction). The possibility of nonlinear associations was tested by generalized additive models (GAMs) indicating linear relationships for all exposure-outcomes associations.

Multivariable log-Poisson regression models with robust standard errors were used for dichotomous outcomes, as these are more appropriate than logistic regression when the incidence of the outcome is 10% or more [34]. Linear regression models were performed for continuous outcomes. Estimated associations were described as relative risks (RR) with 95% confidence intervals (CIs) or β-coefficients with 95% CIs accordingly. We examined the associations of maternal metabolic profile in early pregnancy with childhood cardiometabolic traits at 4 years of age in 3 models: The first model (crude model) was adjusted for the child’s sex (except models using offspring systolic and diastolic blood pressure percentiles as an outcome); the second model (confounder model) was additionally adjusted for maternal age, education level, parity, smoking during pregnancy and pre-pregnancy BMI (only for models using maternal fasting lipid levels or blood pressure as an exposure variable). In a third model (mediation model), we additionally adjusted for maternal weight gain during pregnancy, birth weight, breastfeeding duration, child’s anthropometry at age of outcome assessment, and child lifestyle characteristics [TV viewing (hours/day)]. Because relations of pre-pregnancy BMI with offspring cardiometabolic traits could be moderated by paternal BMI we also examined associations after adjusting for paternal BMI.

We examined potential effect modification by child’s sex, maternal smoking during pregnancy, and gestational weight gain by including the interaction term in the models (statistically significant effect modification if p-value<0.05) and stratified analyses accordingly. We also examined potential effect modification by child BMI in the models using child lipid levels as an outcome variable.

All hypotheses testing were conducted assuming a 0.05 significance level and a 2-sided alternative hypothesis. We used Stata S.E. version 11.2 for the statistical analyses (Stata Corp, Texas, USA).

### Results

#### Participants’ characteristics

Maternal and child demographic characteristics according to maternal overweight/obesity status are shown in Table 1. A total of 209 (34%) women were overweight/obese pre-pregnancy, while 77 (12.5%) women were obese prior to gestation. Overweight/obese women prior to gestation were more likely to be multiparous, less educated, to gain excessive weight during pregnancy and to breastfeed their children for shorter durations compared with women with no excess weight (Table 1). S1 Table in the supporting information material shows that mothers without offspring follow-up data were more likely to be younger, smokers, less educated, and of non-Greek ethnicity. There were no significant differences in socio- demographic characteristics between women who provided fasting blood samples and those who did not (S2 Table).

**Table 1:** Mother- child characteristics by pre-pregnancy overweight/obesity status, Rhea pregnancy cohort, Crete, Greece.

	Pre-pregnancy obesity status		
	No excess weight	Overweight/Obese	*P*- value ^a^
	(n = 409)	(n = 209)	
**Maternal characteristics**			
Maternal age at delivery (yr), *mean (SD)*	29.87 (0.2)	29.98 (0.3)	0.891
Education, *n (%)*			<0.001
*Low*	49 (12.0)	54 (25.8)	
*Medium*	208 (50.9)	102 (48.8)	
*High*	152 (37.2)	53 (25.4)	
Greek origin, *n (%)*	384 (93.9)	199 (95.2)	0.499
Primiparous, *n (%)*	194 (47.4)	70 (33.5)	0.001
Smoking during pregnancy, *n (%)*	126 (30.8)	70 (33.5)	0.497
Gestational diabetes, *n (%)*	32 (8.4)	18 (9.2)	0.758
Gestational hypertension, *n (%)*	15 (4.0)	14 (7.1)	0.106
Gestational weight gain (kg), *n (%)*			<0.001
*Inadequate*	114 (27.9)	15 (7.2)	
*Adequate*	155 (37.9)	81 (38.8)	
*Excessive*	140 (34.2)	113 (54.1)	
Caesarian section, *n (%)*	195 (48.0)	115 (55.0)	0.100
**Metabolic profile in early pregnancy (n = 348)**			
Glucose (mg/dL), *mean (SD)*	74.93 (0.7)	76.10 (1.2)	0.341
Insulin (mg/dL), *mean (SD)*	9.33 (0.9)	12.75 (1.5)	<0.001
TC (mg/dL), *mean (SD)*	195.49 (2.3)	203.87 (3.7)	0.025
LDL-C (mg/dL), *mean (SD)*	113.87 (1.8)	123.14 (3.0)	0.008
HDL-C (mg/dL), *mean (SD)*	60.18 (0.9)	55.42 (1.3)	0.007
TG (mg/dL), *mean (SD)*	108.04 (2.8)	126.23 (4.8)	<0.001
SBP (mmHg), *mean (SD)*	105.06 (0.5)	109.92 (0.8)	<0.001
DBP (mmHg), *mean (SD)*	69.27 (0.5)	71.31 (0.7)	0.013
**Child characteristics in infancy**			
Sex, girl, *n (%)*	201 (49.1)	93 (44.5)	0.274
Birth weight (kg), *mean (SD)*	3.20 (0.02)	3.21 (0.03)	0.887
Gestational age (weeks), *mean (SD)*	38.32 (0.07)	38.08 (0.11)	0.123
Duration of breastfeeding (months), *mean (SD)*	4.69 (0.2)	3.44 (0.3)	<0.001
Day care attendance in the first 2 years of life, *n (%)*	79 (19.5)	36 (17.3)	0.518
**Child characteristics at 4 years of age**			
BMI (Kg/m^2^), *mean (SD)*	16.11 (0.08)	16.93 (0.15)	<0.001
Overweight/obese, *n (%)*	74 (18.1)	60 (28.7)	0.002
Waist circumference (cm), *mean (SD)*	52.90 (0.2)	54.66 (0.4)	<0.001
Waist circumference ≥90th pct, *n (%)*	35 (8.7)	37 (17.9)	<0.001
Sum of skinfolds (mm), *mean (SD)*	37.78 (0.7)	43.29 (1.1)	<0.001
TC (mg/dL), *mean (SD)*	156.39 (1.5)	158.97 (2.0)	0.247
LDL-C (mg/dL), *mean (SD)*	69.98 (27.4)	69.58 (26.7)	0.856
HDL-C (mg/dL), *mean (SD)*	47.17 (0.6)	47.78 (0.8)	0.518
TG (mg/dL), *mean (SD)*	69.69 (1.5)	70.40 (2.1)	0.690
Time spent watching TV (hours/day), *n (%)*			0.019
*Almost never*	124 (30.5)	52 (25.0)	
*1–2 hours/day*	251 (61.8)	126 (60.6)	
*More than 3 hours/day*	31 (7.6)	30 (14.4)	
Energy intake (Kcals/day), *mean (SD)*	1583.5 (23.0)	1594.3 (32.1)	0.752

BMI, Body Mass Index; TC, Total Cholesterol; LDL-C, Low Density Lipoprotein Cholesterol; HDL-C, High Density

Lipoprotein Cholesterol; TG, Triglyceride; CRP, C-reactive protein; SBP, Systolic Blood Pressure; DBP, Diastolic Blood Pressure; pct, percentile

^**a**^ *P*-values obtained by Mann-Whitney U test for two independent samples, and χ^2^ test or Fisher exact test with Monte-Carlo correction.

Numbers may not correspond to the total due to missing numbers.

In the subset of pregnant women with available fasting serum samples in early pregnancy, dyslipidemia was the most frequent metabolic disorder, as 49.7% women had total cholesterol levels ≥ 200 mg/dL, 26.8% had HDL-C levels< 50 mg/dL, and 18.7% had TG levels ≥ 150 mg/dL. Only 22 (6.3%) women were suffering from hyperglycemia in the first trimester of pregnancy. Overweight/obesity prior to gestation was associated with higher fasting total cholesterol, LDL-C, triglycerides, and insulin levels at the first trimester of pregnancy, lower HDL-C levels, and higher systolic and diastolic blood pressure (Table 1).

The prevalence of overweight/obesity and central adiposity (WC ≥ 90th percentile) at 4 years of age was 21.7% (n = 134) and 11.8% (n = 72), respectively (Table 1). Mean (±SD) non fasting total cholesterol, LDL-C and HDL-C were 198.1 (±36.2), 116.7 (±29.4), and 58.7 (±14.1) respectively. Children whose mothers were overweight/obese prior to gestation had higher BMI, waist circumference, and fat mass at 4 years of age compared to children whose mothers had no excess weight pre-pregnancy (Table 1).

### Overweight/obesity pre-pregnancy in association with offspring cardiometabolic traits at 4 years of age

**Fig 1:**
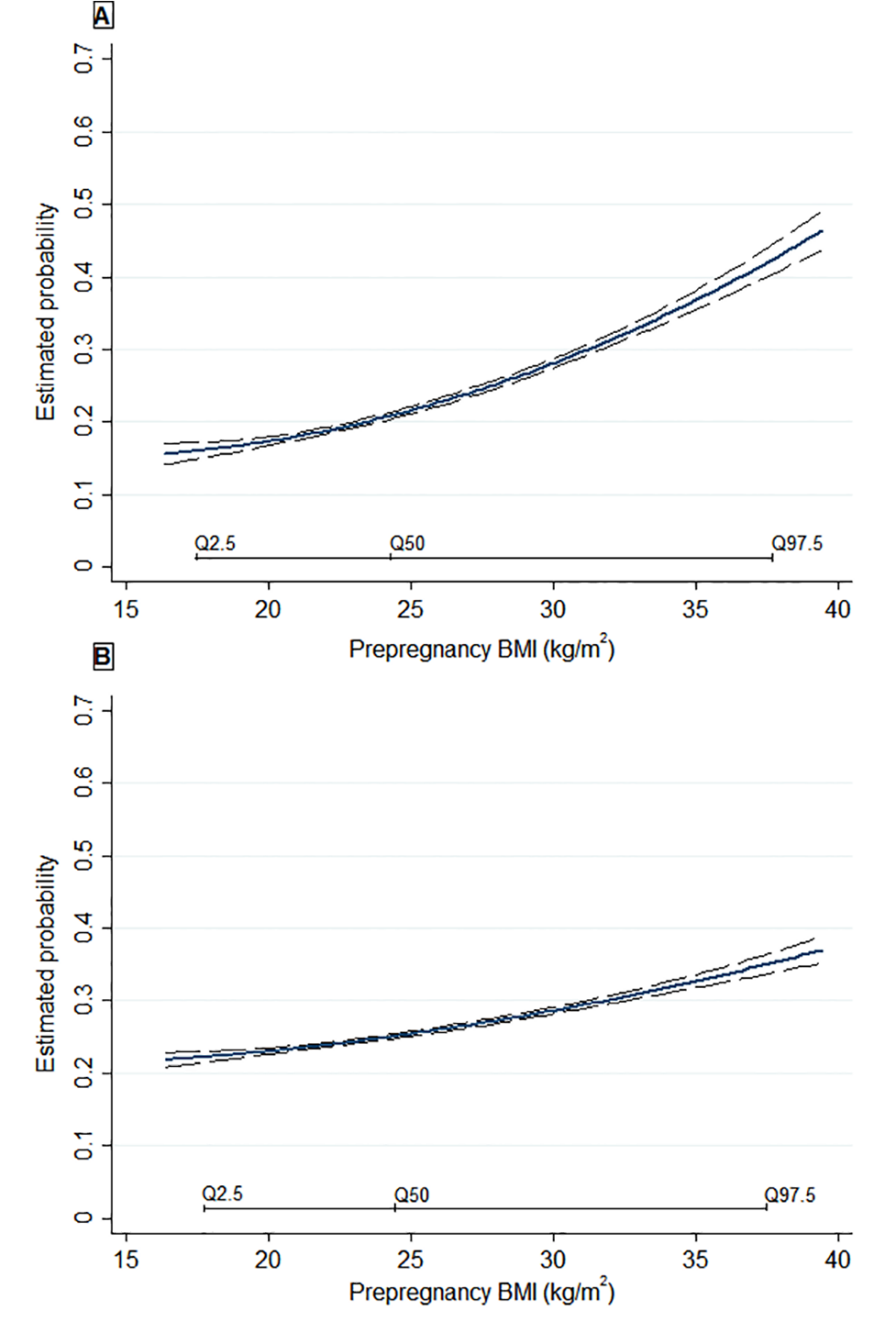
Relationship between pre-pregnancy BMI and the estimated probability for overweight/obesity (A) and cholesterol levels ≥75th percentile (B) at 4 years of age. Estimated probability is based on multivariable models adjusted for maternal age, education, parity, smoking during pregnancy, gestational weight gain, birth weight, breastfeeding duration and TV watching at 4 years of age. Q2.5, Q50, Q97.5 represent the 2.5th, 50.0th, and the 97.5th percentiles of the studied population. Long-dashes represent the 95%CIs.

Generalised additive models examining the shape of the relationships of metabolic profile in early pregnancy with offspring cardiometabolic traits at 4 years of age showed no significant departures from linearity overall. Pre-pregnancy overweight/obesity showed a positive linear relationship with the probability of overweight/obesity at 4 years of age (Fig 1). Pre-pregnancy overweight/obesity was also positively associated with all other offspring adiposity outcomes at 4 years of age (Table 2). We found no association between pre-pregnancy BMI and offspring non-fasting lipid levels or blood pressure percentiles at 4 years of age (Table 2). Further adjustment for paternal BMI did not attenuate the observed associations (S3 Table).

**Table 2:** Association of maternal pre-pregnancy obesity status with offspring cardiometabolic traits at 4 years of age, Rhea pregnancy cohort Crete, Greece.

		Pre-pregnancy overweight/obese (≥25 kg/m^2^)		
		(n = 209)		
Cardiometabolic traits at 4 years of age	n	Model 1	Model 2	Model 3
*Adiposity outcomes*				
		RR (95%CI)	RR (95%CI)	RR (95%CI)
Overweight/obese	134	**1.59 (1.17, 2.15)**	**1.53 (1.11, 2.09)**	**1.83 (1.19, 2.81)**
WC (cm) ≥ 90th pct	72	**2.03 (1.32, 3.14)**	**1.89 (1.20, 2.96)**	**1.97 (1.11, 3.49)**
		**β-coef. (95%CI)**	**β-coef. (95%CI)**	**β-coef. (95%CI)**
Child BMI	618	**0.80 (0.45, 1.14)**	**0.78 (0.44, 1.12)**	**0.79 (0.36, 1.06)**
WC (cm)	606	**1.75 (0.87, 2.63)**	**1.76 (0.89, 2.64)**	**1.36 (0.55, 2.17)**
Sum of 4 Skinfolds (mm)	601	**5.74 (3.17, 8.30)**	**5.37 (2.75, 7.99)**	**5.10 (2.49, 7.71)**
***Non-fasting lipid levels***		**β-coeff. (95%CI)**	**β-coef. (95%CI)**	**β-coef. (95%CI)**
TC(mg/dl)	525	2.64 (-2.21, 7.50)	2.52 (-2.50. 7.55)	2.18 (-3.04. 7.41)
HDL-C(mg/dl)	525	0.59 (-1.38, 2.56)	0.43 (-1.63, 2.49)	0.59 (-1.54, 2.73)
***Blood pressure levels***		**β-coef. (95%CI)**	**β-coef. (95%CI)**	**β-coef. (95%CI)**
SBP percentiles	488	0.26 (-0.17, 0.69)	0.30 (-0.13, 0.75)	0.21 (-0.24, 0.67)
DBP percentiles	488	-0.07 (-0.49, 0.34)	-0.11 (-0.53, 0.31)	-0.10 (-0.54, 0.33)

BMI, Body Mass Index; WC, Waist Circumference; TC, Total Cholesterol; LDL-C, Low Density Lipoprotein Cholesterol; HDL-C, High Density Lipoprotein Cholesterol; SBP, Systolic Blood Pressure; DBP, Diastolic Blood Pressure; pct, percentile

Model 1: adjusted for child sex. (except models using offspring systolic and diastolic blood pressure percentiles as an outcome)

Model 2: model 1 further adjusted for maternal age, education level, parity, smoking during pregnancy

Model 3: model 2 additionally adjusted for gestational weight gain, birth weight, breastfeeding duration, and TV watching at 4

years of age (hours/day). Models using offspring WC and sum of skinfolds as an outcome variable were also adjusted for child

height, while those using offspring non-fasting lipid levels as an outcome were also adjusted for child BMI. Bold indicated statistically significant differences at p<0.05.

### Fasting lipid, glucose and insulin levels in early pregnancy in association with offspring cardiometabolic traits at 4 years of age

**Fig 2:**
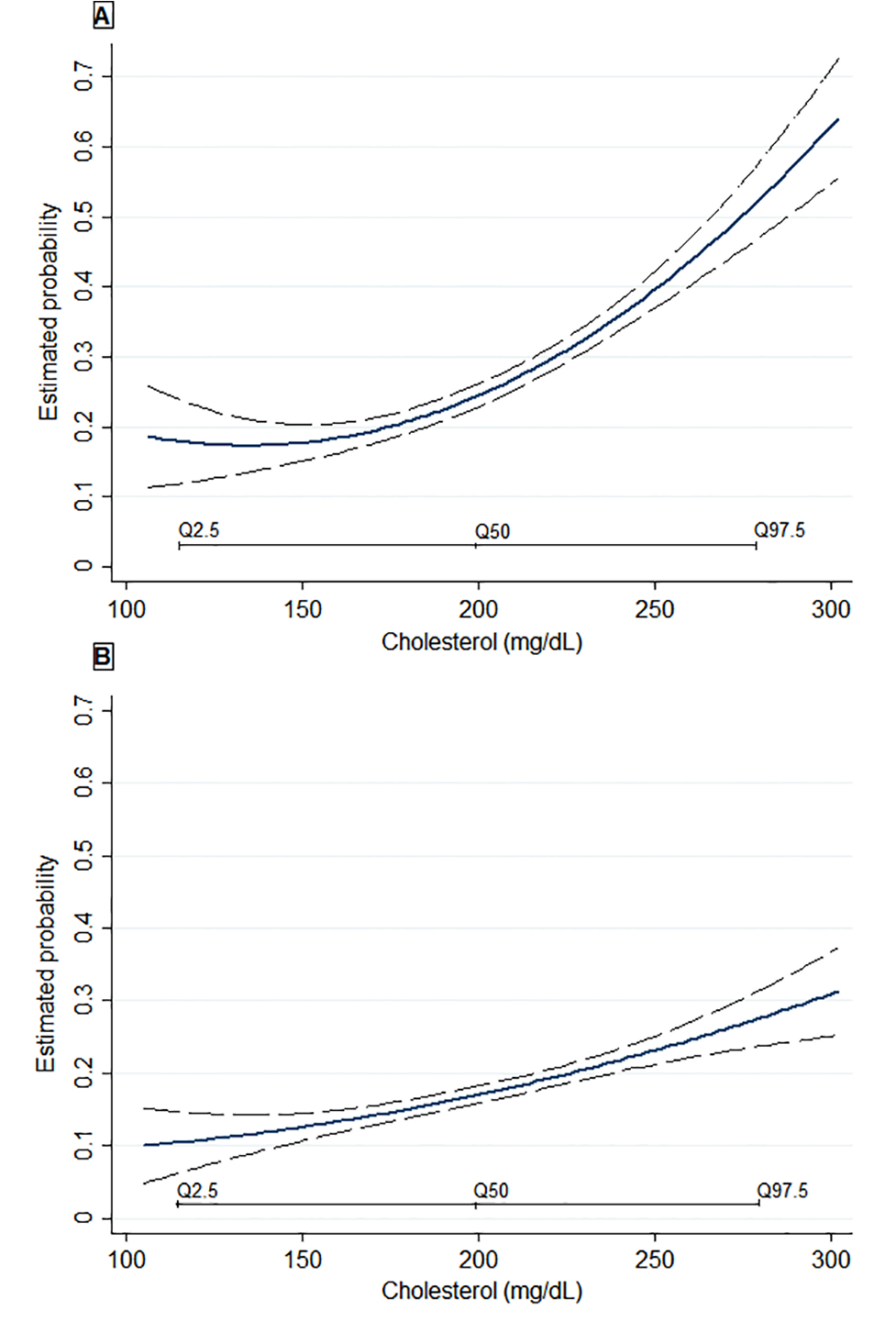
Relationship between first-trimester fasting maternal cholesterol levels and the estimated probability for overweight/obesity (A) and cholesterol levels ≥75th percentile (B) at 4 years of age. Estimated probability is based on multivariable models adjusted for maternal age, education, parity, smoking during pregnancy, BMI pre-pregnancy, gestational weight gain, birth weight, breastfeeding duration, and TV watching at 4 years of age (hours/day). Q2.5, Q50, Q97.5 represent the 2.5th, 50.0th, and the 97.5th percentiles of the studied population. Long-dashes represent the 95%CIs.

Maternal fasting cholesterol levels showed a positive linear relationship with the probability of overweight/obesity at 4 years of age (Fig 2). An increase of 40mg/dl in total cholesterol levels was associated with 42% increased risk of overweight/obesity (RR: 1.42, 95% CI: 1.03, 1.95) and greater skinfold thickness by 3.30 mm (95%CI: 1.41, 5.20) at 4 years of age after adjustment for several covariates and pre-pregnancy BMI (Model 3). A positive association was also observed between maternal fasting cholesterol levels and offspring cholesterol levels at 4 years of age, but the associations were attenuated when we further adjusted for potential mediators (Table 3, model 3). No association was found between maternal fasting cholesterol levels and offspring blood pressure percentiles at 4 years of age (Table 3). No association was also observed among maternal fasting glucose and insulin serum levels in early pregnancy and offspring cardiometabolic traits at 4 years of age (data not shown).

**Table 3:** Association of maternal fasting lipid profile in early pregnancy with offspring cardiometabolic traits at 4 years of age, Rhea pregnancy cohort Crete, Greece.

		Fasting TC levels in early pregnancy			Fasting LDL-C levels in early pregnancy		
		(per increase in 40 mg/dL)			(per increase in 15 mg/dL)		
		(n = 348)			(n = 348)		
Cardiometabolic traits at 4 years of age	n	Model 1	Model 2	Model 3	Model 1	Model 2	Model 3
*Adiposity outcomes*							
		**RR (95%CI)**	**RR (95%CI)**	**RR (95%CI)**	**RR (95%CI)**	**RR (95%CI)**	**RR (95%CI)**
Overweight/obese	64	1.18 (0.92, 1.50)	1.27 (0.97, 1.66)	**1.42 (1.03, 1.95)**	1.03 (0.91, 1.17)	1.07 (0.93, 1.22)	1.10 (0.94, 1.29)
WC (cm) ≥ 90th pct	30	1.07 (0.73, 1.56)	1.07 (0.71, 1.62)	1.24 (0.74, 2.05)	1.02 (0.84, 1.23)	1.07 (0.87, 1.32)	1.12 (0.88, 1.44)
		**β-coef. (95%CI)**	**β-coef. (95%CI)**	**β-coef. (95%CI)**	**β-coef. (95%CI)**	**β-coef. (95%CI)**	**β-coef. (95%CI)**
Child BMI	348	0.08 (-0.12, 0.29)	0.04 (-0.18, 0.26)	0.01 (-0.24, 0.26)	0.02 (-0.08, 0.13)	0.00 (-0.12, 0.13)	-0.01 (-0.16, 0.13)
WC (cm)	348	0.34 (-0.26, 0.95)	0.29 (-0.41, 1.00)	0.40 (-0.25, 1.07)	0.34 (-0.26, 0.95)	0.29 (-0.41, 1.00)	0.40 (-0.25, 1.07)
Sum of 4 Skinfolds (mm)	341	**2.53 (0.92, 4.14)**	**2.76 (1.00, 4.52)**	**3.30 (1.41, 5.20)**	0.77 (-0.08, 1.63)	0.80 (-0.16, 1.77)	**1.11 (0.08, 2.13)**
***Non-fasting lipid levels***		**β-coef. (95%CI)**	**β-coeff. (95%CI)**	**β-coeff. (95%CI)**	**β-coeff. (95%CI)**	**β-coeff. (95%CI)**	**β-coeff. (95%CI)**
TC(mg/dl)	294	**4.14 (1.00, 7.28)**	**3.43 (0.04, 6.83)**	3.25 (-0.53, 7.04)	**2.09 (0.56, 3.61)**	**1.93 (0.28, 3.58)**	1.90 (-0.04, 3.85)
HDL-C(mg/dl)	294	-0.18 (-1.47, 1.11)	-0.59 (-2.06, 0.87)	-1.04 (-2.74, 0.65)	-0.35 (-0.95, 0.24)	-0.56 (-1.24, 0.10)	-0.71 (-1.49, 0.06)
***Blood pressure levels***		**β-coef. (95%CI)**	**β-coef. (95%CI)**	**β-coef. (95%CI)**	**β-coef. (95%CI)**	**β-coef. (95%CI)**	**β-coef. (95%CI)**
SBP percentiles	284	-0.05 (-0.35, 0.24)	-0.07 (-0.40, 0.25)	-0.06 (-0.42, 0.29)	-0.04 (-0.19, 0.09)	-0.05 (-0.22, 0.10)	-0.06 (-0.24, 0.10)
DBP percentiles	284	-0.19 (-0.50, 0.12)	-0.19 (-0.52, 0.12)	-0.15 (-0.51, 0.21)	-0.08 (-0.23, 0.06)	-0.09 (-0.24, 0.06)	-0.09 (-0.27, 0.07)

BMI, Body Mass Index; WC, Waist Circumference; TC, Total Cholesterol; LDL-C, Low Density Lipoprotein Cholesterol; HDL-C, High Density Lipoprotein Cholesterol; SBP, Systolic Blood Pressure; DBP, Diastolic Blood Pressure; pct, percentile

Model 1: adjusted for child sex. (except models using offspring systolic and diastolic blood pressure percentiles as an outcome)

Model 2: model 1 further adjusted for maternal age, education level, parity, smoking during pregnancy and pre-pregnancy BMI

Model 3: model 2 additionally adjusted for gestational weight gain, birth weight, breastfeeding duration, and TV watching at 4 years of age (hours/day). Models using offspring WC and sum of skinfolds as an outcome variable were also adjusted for child height, while those using offspring non-fasting lipid levels as an outcome were also adjusted for child BMI. Bold indicated statistically significant differences at p<0.05

### Maternal blood pressure levels in early pregnancy in association with offspring cardiometabolic traits at 4 years of age

An increase of 10mmHg in diastolic blood pressure in early pregnancy was associated with 23% increased risk of offspring overweight/obesity (RR: 1.22, 95%CI: 1.03, 1.45), and greater skin fold thickness by 1.71 mm (95%CI: 0.57, 2.86) in the fully adjusted model (Table 4). We found no association between maternal blood pressure levels in early pregnancy and offspring lipids and blood pressure percentiles at 4 years of age (Table 4).

**Table 4:** Association of maternal blood pressure levels in early pregnancy with offspring cardiometabolic traits at 4 years of age, Rhea pregnancy cohort Crete, Greece.

		SBP in early pregnancy			DBP in early pregnancy		
		(per increase in 10 mm Hg)			(per increase in 10 mm Hg)		
		(n = 536)			(n = 536)		
Cardiometabolic traits at 4 years of age	n	Model 1	Model 2	Model 3	Model 1	Model 2	Model 3
*Adiposity outcomes*							
		**RR (95%CI)**	**RR (95%CI)**	**RR (95%CI)**	**RR (95%CI)**	**RR (95%CI)**	**RR (95%CI)**
Overweight/obese	156	**1.18 (1.01, 1.37)**	1.11 (0.95, 1.31)	1.15 (0.97, 1.36)	**1.23 (1.05, 1.45)**	**1.19 (1.01, 1.41)**	**1.22 (1.03, 1.45)**
WC (cm) ≥ 90th pct	60	1.16 (0.93, 1.44)	1.02 0.81, 1.29)	1.03 (0.82, 1.31)	1.19 (0.94, 1.50)	1.09 (0.84, 1.40)	1.09 (0.84, 1.41)
		**β-coeff. (95%CI)**	**β-coeff. (95%CI)**	**β-coeff. (95%CI)**	**β-coeff. (95%CI)**	**β-coeff. (95%CI)**	**β-coeff. (95%CI)**
Child BMI	536	0.15 (-0.00, 0.30)	0.05 (-0.10, 0.21)	0.09 (-0.06, 0.26)	0.13 (-0.03, 0.29)	0.06 (-0.09, 0.23)	0.09 (-0.06, 0.25)
WC (cm)	532	**0.38 (0.01, 0.76)**	0.20 (-0.18, 0.58)	0.22 (-0.12, 0.57)	0.36 (-0.05, 0.79)	0.21 (-0.19, 0.62)	0.26 (-0.09, 0.61)
Sum of 4 Skinfolds (mm)	524	1.59 (0.35, 2.83)	0.98 (-0.32, 2.30)	1.11 (-0.19, 2.42)	**1.96 (0.79, 3.13)**	**1.49 (0.32, 2.66)**	**1.71 (0.57, 2.86)**
***Non-fasting lipid levels***		**β-coeff. (95%CI)**	**β-coeff. (95%CI)**	**β-coeff. (95%CI)**	**β-coeff. (95%CI)**	**β-coeff. (95%CI)**	**β-coeff. (95%CI)**
TC(mg/dl)	458	-0.33 (-2.50, 1.83)	-0.81 (-3.05, 1.42)	-0.97 (-3.22, 1.27)	-0.85 (-3.50, 1.79)	-1.03 (-3.70, 1.63)	-1.12 (-3.86, 1.60)
HDL-C(mg/dl)	458	0.64 (-0.26, 1.54)	0.80 (-0.16, 1.77)	0.80 (-0.20, 1.80)	-0.21 (-1.23, 0.80)	0.04 (-1.00, 1.10)	-0.02 (-1.11, 1.05)
***Blood pressure levels***		**β-coeff. (95%CI)**	**β-coeff. (95%CI)**	**β-coeff. (95%CI)**	**β-coeff. (95%CI)**	**β-coeff. (95%CI)**	**β-coeff. (95%CI)**
SBP percentiles	422	0.05 (-0.12, 0.22)	0.04 (-0.15, 0.23)	0.08 (-0.10, 0.27)	0.10 (-0.10, 0.31)	0.08 (-0.13, 0.30)	0.07 (-0.14, 0.29)
DBP percentiles	422	0.00 (-0.18, 0.19)	0.04 (-0.14, 0.24)	0.02 (-0.16, 0.22)	0.08 (-0.12, 0.29)	0.06 (-0.14, 0.27)	0.07 (-0.15, 0.29)

BMI, Body Mass Index; WC, Waist Circumference; TC, Total Cholesterol; LDL-C, Low Density Lipoprotein Cholesterol; HDL-C, High Density Lipoprotein Cholesterol; SBP, Systolic Blood Pressure; DBP, Diastolic Blood Pressure; pct, percentile

Model 1: adjusted for child sex. (except models using offspring systolic and diastolic blood pressure percentiles as an outcome)

Model 2: model 1 further adjusted for maternal age, education level, parity, smoking during pregnancy and pre-pregnancy BMI

Model 3: model 2 additionally adjusted for gestational weight gain, birth weight, breastfeeding duration, and TV watching at 4 years of age (hours/day). Models using offspring WC and sum of skinfolds as an outcome variable were also adjusted for child height, while those using offspring non-fasting lipid levels as an outcome were also adjusted for child BMI. Bold indicated statistically significant differences at *p*<0.05

### Effect modification-Sensitivity analyses

In the mediation model, further adjustment for birth characteristics, child's anthropometry and life-style behaviors did not substantively alter the adjusted estimations for most childhood outcomes (Tables 2, 3 and 4). Further analyses showed evidence for an interaction between child sex and maternal pre-pregnancy BMI in response to offspring overweight/obesity and central adiposity (p for interaction <0.05), but not with skinfold thickness (Table 5). The greatest risk for these adiposity outcomes was observed for girls whose mothers were overweight/obese prior to gestation (RR-overweight/obesity: 3.54, 95%CI: 1.80, 6.98; RR-central adiposity: 5.33, 95%CI: 2.17, 13.07), whereas similar associations in boys were not significant (Table 5). We saw no evidence for a multiplicative interaction of maternal metabolic profile in early pregnancy with maternal smoking during pregnancy, gestational weight gain or child BMI (p for interaction >0.05).

**Table 5:** Association of pre-pregnancy BMI with offspring obesity measures at 4 years of age, stratified by child sex, Rhea pregnancy cohort, Crete, Greece.

	Offspring adiposity measures at 4 years of age		
	Overweight/obese	WC (cm) ≥ 90th percentile ^a^	Sum of 4 skinfolds (mm)^a^
	(n = 134)	(n = 72)	(n = 601)
	RR (95% CI)	RR (95% CI)	β-coeff.(95% CI)
**Maternal pre-pregnancy BMI (kg/m** ^**2**^ **)**			
All *(n = 618)*	**1.08 (1.04, 1.13)**	**1.10 (1.04, 1.16)**	**0.51 (0.25, 0.77)**
Boys *(n = 324)*	1.04 (0.98, 1.10)	1.02 (0.93, 1.11)	0.22 (-0.07, 0.52)
Girls *(n = 294)*	**1.13 (1.06, 1.20)**	**1.19 (1.10, 1.29)**	**0.79 (0.34, 1.25)**
*P for interaction*	**0.032**	**0.004**	**0.030**
**Overweight/Obese (≥25kg/m** ^**2**^ **) prior to gestation**			
All *(n = 209)*	**1.83 (1.19, 2.81)**	**1.97 (1.11, 3.49)**	**5.10 (2.49, 7.71)**
Boys *(n = 116)*	1.10 (0.61, 2.01)	0.97 (0.42, 2.21)	**3.03 (0.07, 5.99)**
Girls *(n = 93)*	**3.54 (1.80, 6.98)**	**5.33 (2.17, 13.07)**	**7.59 (3.10, 12.08)**
*P for interaction*	**0.007**	**0.007**	0.061

BMI, Body Mass Index; WC, Waist Circumference

All models are adjusted for maternal age, education, parity, smoking during pregnancy, gestational weight gain, birth weight, breastfeeding duration, and

TV watching at 4 years of age (hours/day).

^a^Also adjusted for child height.

Bold indicated statistically significant differences at p<0.05.

To elucidate whether gestational diabetes modified the observed results, we performed a sensitivity analysis in which we excluded all women who were diagnosed with gestational diabetes (n = 50). Results did not differ substantially from those derived from the main analysis (S4 Table, S5 Table and S6 Table). We also found no difference in the observed estimates after excluding preterm births (data not shown).

## Discussion

In this prospective pregnancy cohort we showed that exposure to metabolic dysregulation in early pregnancy may predict increased risk of obesity in preschool children. To our knowledge this is the first study examining maternal metabolic profile in early pregnancy with the use of fasting serum samples in association with offspring cardiometabolic risk.

Our findings are in consistence with previous epidemiological studies examining the effect of maternal pre-pregnancy BMI with child BMI measures [7, 8, 35], and fat mass [12, 14, 22, 36, 37]. The associations of maternal pre-pregnancy BMI with childhood adiposity may be explained by intrauterine mechanisms or shared environmental, life-style and genetic characteristics [9]. Animal studies suggest that epigenetic alterations induced by maternal overnutrition in pregnancy may modulate expression of genes that regulate adipogenesis, glucose homeostasis, inflammation, and/or insulin signaling, including genes encoding hormones (e.g., leptin), nuclear receptors (adipogenic and lipogenic transcription factors PPARγ and PPARα, respectively), gluconeogenic enzymes and transmembrane proteins [38]. Moreover, adverse maternal conditions such as maternal obesity, have been demonstrated in animal studies to affect placental morphology, blood flow, feto-maternal exchanges and endocrine function, which have direct consequences for fetal tissue development, and may lead to a higher offspring susceptibility to develop metabolic disorders [39]. Although adjustment for several sociodemographic and lifestyle related characteristics did not explain our findings, we cannot rule out the possibility of residual confounding mainly related to shared mother-child lifestyle. The observed effects were not mediated by pregnancy complications such as gestational diabetes, birth characteristics, and infant feeding patterns (breastfeeding), which have been associated with both maternal BMI and offspring postnatal growth. Additionally, our results remained substantially the same after adjusting for paternal BMI, implying potential intrauterine mechanisms in the observed associations.

The greatest risk for overweight/obesity and central adiposity was observed for girls whose mothers were overweight/obese prior to gestation. The long-term effects of the same environmental insult, such as maternal obesity, can have various phenotypic effects on male and female offspring [17]. There are no consistent findings from epidemiological studies on offspring sex-specific responses to maternal weight status, while sex specificity in response to maternal anthropometry has been shown in fetal growth measures [40]. Animal studies have shown that there are sex-specific differences in the regulation and expression of placental genes, proteins, steroids and structure [41, 42]. Microarray animal experiments also showed that the response to a high-fat diet during gestation triggers sex-specific epigenetic alterations throughout the genome, together with sexually dimorphic deregulation of clusters of imprinted genes: mainly cell signaling involving immune cells, and uptake and metabolism of amino acids for females, and development and function of the vascular system, and uptake and metabolism of glucose and fatty acids for males [43]. Timing of exposure is also a critical issue in sex differences in developmental programming. Female fetuses respond more than males to the mother’s preconception nutrition and metabolism, while, in contrast, male fetuses are more vulnerable to metabolic changes during gestation, especially after the first trimester of pregnancy [44, 45]. Further epidemiological studies are needed to explore the sex-specific causal variables and how females versus males respond and adapt to maternal obesity.

To our knowledge, this is the first study to investigate associations of maternal blood pressure levels at the first trimester of pregnancy with offspring cardiometabolic traits. We found that high blood pressure levels in early pregnancy were associated with increased risk of overweight/obesity and increased fat mass at 4 years of age after excluding pre-eclamptic pregnancies. The observed effects were not attenuated by pre-pregnancy BMI or weight gain during pregnancy, implying an independent role of maternal blood pressure to offspring fat distribution. Maternal hypertension in early pregnancy may disrupt the typical physiological changes in the spiral arteries of the decidua and myometrium, resulting in poor placental perfusion, early placental hypoxia and oxidative stress [46]. Therefore, it may be possible that even a small increase in blood pressure levels (10mmHg) in early pregnancy may predispose to adverse metabolic outcomes and increased fat mass later in life.

We also found that abnormal fasting cholesterol levels in early pregnancy were associated with increased risk of offspring overweight/obesity, and greater fat mass at 4 years of age. The observed associations were not attenuated by maternal BMI pre-pregnancy, gestational diabetes status, birth size, or child BMI at age of outcome assessment. Gademan et al reported recently a positive association between maternal lipid levels and offspring fat percentage and waist-to height ratio values at 5–6 years of age children [22]. There are no other studies so far on the association of maternal lipid levels in pregnancy with offspring cardiometabolic traits other than adiposity measures. Maternal dyslipidemia could increase the oxidative stress in the fetus resulting not only in damage of the vessel wall, but also in the disruption of normal placentation. Hypercholesterolemia in early pregnancy has been associated with increased offspring atherosclerotic lesions both in animal models [47], and in human tissues [48]. One potential explanation for such increased cardiometabolic risk in children of mothers with hypercholesterolemia, is the induction of a constitutional state of overexpression of “atherogenes” in the fetal vascular wall by maternal hypercholesterolemia or the resulting fatty-streak formation [48].

### Strengths and limitations

The strengths of the present study include the population-based prospective design and detailed cardiometabolic measurements in early pregnancy and in childhood. Unlike previous epidemiologic studies, blood pressure, lipids, glucose, and insulin concentrations were not collected from medical records but measured during the study follow up according to validated protocols. Moreover, fasting serum samples were available in early pregnancy that is a rather complicated goal for a cohort involving pregnant women. The exclusion of mother-child pairs with multiple pregnancies, pregnancies with preeclampsia, as well as adjustment for several sociodemographic variables, reduced the likelihood of potential confounding. However, because of the observational study design, residual confounding because of other unmeasured confounders may still occur.

The levels of attrition in the Rhea cohort is similar to those found in other birth cohort studies. Offspring of more educated women, and of older women were more likely to attend follow-up clinical assessment. Assuming that mothers and children with a higher BMI are less likely to participate in a detailed obesity follow-up, our estimates may be underestimated. A selection bias could be theoretically generated by the possibility that we included only women receiving an early ultrasound. However, all pregnant women in Greece have to attend several compulsory prenatal visits, one of which take place around 12 weeks of gestation, which is the time of our enrolment phase. Information on maternal pre-pregnancy weight was self-reported, which might have led to misclassification of maternal BMI pre-pregnancy. However, we have performed a validation study comparing self-reported pre-pregnancy weight with clinically measured weight in the first prenatal visit, which was available in our cohort, showing high correlation (r 0.93) and a fairly good agreement between self-reported and objectively measured BMI (Bland-Altman plots, data not shown). We were not able to measure fasting lipid levels to children at 4 years follow up, as expected at this age of follow up. It has been shown that children fasting serum lipids levels have small differences with non-fasting levels[49].

## Conclusions

The results of the present study indicate that metabolic dysregulation in early pregnancy may determine offspring obesity at preschool age. The complex underlying mechanisms that explain these findings require additional study. Further follow-up of this cohort will allow to determine whether maternal metabolic profile in early pregnancy is associated with a broader range of offspring cardiometabolic disorders at later ages.

## Supporting Information

S1 Table Maternal and child characteristics of participants and non-participants in the childhood follow up of the Rhea pregnancy cohort Crete, Greece. ^a^ Statistically significant differences (*p*<0.05), based on Mann-Whitney U test for two independent samples and Pearson's χ2 test for independence.
(PDF)

S2 Table Maternal and child characteristics of women who provided fasting blood samples in early pregnancy and those who did not, Rhea pregnancy cohort Crete, Greece. ^a^ Statistically significant differences (*p*<0.05), based on Mann-Whitney U test for two independent samples and Pearson's χ2 test for independence.
(PDF)

S3 Table Association of maternal pre-pregnancy obesity status with offspring cardiometabolic traits at 4 years of age after adjustment for paternal BMI, Rhea pregnancy cohort Crete, Greece. BMI, Body Mass Index; WC, Waist Circumference; TC, Total Cholesterol; LDL-C, Low Density Lipoprotein Cholesterol; HDL-C, High Density Lipoprotein Cholesterol; SBP, Systolic Blood Pressure; DBP, Diastolic Blood Pressure. All models were adjusted for child sex (except models using offspring systolic and diastolic blood pressure percentiles as an outcome) maternal age, education level, parity, smoking during pregnancy, gestational weight gain, birth weight, breastfeeding duration, TV watching at 4 years of age (hours/day) and paternal BMI. Models using offspring WC and sum of skinfolds as an outcome variable were also adjusted for child height, while those using offspring non-fasting lipid levels as an outcome were also adjusted for child BMI. Bold indicated statistically significant differences at *p*<0.05.
(PDF)

S4 Table Association of maternal pre-pregnancy obesity status with offspring cardiometabolic traits at 4 years of age, after excluding women with gestational diabetes (n = 50), Rhea pregnancy cohort Crete, Greece. BMI, Body Mass Index; WC, Waist Circumference; TC, Total Cholesterol; LDL-C, Low Density Lipoprotein Cholesterol; HDL-C, High Density Lipoprotein Cholesterol; SBP, Systolic Blood Pressure; DBP, Diastolic Blood Pressure; Model 1: adjusted for child sex. (except models using offspring systolic and diastolic blood pressure percentiles as an outcome). Model 2: model 1 further adjusted for maternal age, education level, parity and smoking during pregnancy Model 3: model 2 additionally adjusted for gestational weight gain, birth weight, breastfeeding duration, and TV watching at 4 years of age (hours/day). Models using offspring WC and sum of skinfolds as an outcome variable were also adjusted for child height, while those using offspring non-fasting lipid levels as an outcome were also adjusted for child BMI. Bold indicated statistically significant differences at p<0.05.
(PDF)

S5 Table Association of maternal fasting lipid profile in early pregnancy with offspring cardiometabolic traits at 4 years of age, after excluding women with gestational diabetes (n = 25), Rhea pregnancy cohort Crete, Greece BMI, Body Mass Index; WC, Waist Circumference; TC, Total Cholesterol; LDL-C, Low Density Lipoprotein Cholesterol; HDL-C, High Density Lipoprotein Cholesterol; SBP, Systolic Blood Pressure; DBP, Diastolic Blood Pressure; Model 1: adjusted for child sex. (except models using offspring systolic and diastolic blood pressure percentiles as an outcome) Model 2: model 1 further adjusted for maternal age, education level, parity, smoking during pregnancy and pre-pregnancy BMI. Model 3: model 2 additionally adjusted for gestational weight gain, birth weight, breastfeeding duration, and TV watching at 4 years of age (hours/day). Models using offspring WC and sum of skinfolds as an outcome variable were also adjusted for child height, while those using offspring non-fasting lipid levels as an outcome were also adjusted for child BMI. Bold indicated statistically significant differences at p<0.05
(PDF)

S6 Table Association of maternal blood pressure levels in early pregnancy with offspring cardiometabolic traits at 4 years of age, after excluding women with gestational diabetes (n = 50), Rhea pregnancy cohort Crete, Greece. BMI, Body Mass Index; WC, Waist Circumference; TC, Total Cholesterol; LDL-C, Low Density Lipoprotein Cholesterol; HDL-C, High Density Lipoprotein Cholesterol; SBP, Systolic Blood Pressure; DBP, Diastolic Blood Pressure;. Model 1: adjusted for child sex (except models using offspring systolic and diastolic blood pressure percentiles as an outcome). Model 2: model 1 further adjusted for maternal age, education level, parity, smoking during pregnancy and pre-pregnancy BMI. Model 3: model 2 additionally adjusted for gestational weight gain, birth weight, breastfeeding duration, and TV watching at 4 years of age (hours/day). Models using offspring WC and sum of skinfolds as an outcome variable were also adjusted for child height, while those using offspring non-fasting lipid levels as an outcome were also adjusted for child BMI. Bold indicated statistically significant differences p<0.05.
(PDF)
