# Supplementary material for: Metabolic Profile in Early Pregnancy Is Associated with Offspring Adiposity at 4 Years of Age: The Rhea Pregnancy Cohort Crete, Greece
Source: PLoS One. 2015 May 13;10(5):e0126327. doi: 10.1371/journal.pone.0126327 (PMC4430416; doi:10.1371/journal.pone.0126327)
Supplement: S1 Table — a Statistically significant differences (p<0.05), based on Mann-Whitney U test for two independent samples and Pearson's χ2 test for independence. (PDF) [file pone.0126327.s001.pdf]

S1 Table. Maternal and child characteristics of participants and non-participants in the childhood follow up of the Rhea pregnancy cohort Crete, Greece

	**Participants (n=698)**	**Non participants (n=610)**	
**Maternal characteristics**			***P*- value[a]**
Maternal age (years), *mean(SD)*	29.83±4.8	28.88±5.2	<0.001
Maternal education, *n (%)*			<0.001
Low	117 (16.8)	153 (26.5)	
Medium	351 (50.3)	294 (51.0)	
High	230 (33.0)	130 (22.5)	
Mother's origin, *n (%)*			<0.001
Greek	660 (94.7)	516 (86.1)	
Non greek	37 (5.3)	83 (13.9)	
Smoking status, *n (%)*			<0.001
Smoker	218 (31.2)	226 (41.0)	
Non-smoker	480 (68.8)	325 (59.0)	
Parity, *n (%)*			0.065
Primiparous	304 (43.6)	217 (38.4)	
Multiparous	394 (56.4)	348 (61.6)	
Pre-pregnancy BMI (kg/m^2), *mean(SD)*	24.51±4.8	23.69±4.6	<0.001
Pre-pregnancy BMI categories			0.284
< 25 kg/m^2	471 (67.5)	402 (70.3)	
≥ 25 kg/m^2	227 (32.5)	170 (29.7)	
Gestational weight gain (kg), *n (%)*			0.210
Inadequate	130 (22.1)	106 (25.2)	
Adequete	203 (34.5)	124 (29.5)	
Excessive	256 (43.50	191 (45.4)	
Delivery type, *n (%)*			0.888
Vaginal	350 (50.4)	303 (50.8)	

Caesarean	345 (49.6)	294 (49.2)	
**Child characteristics**			
Child gender, *n (%)*			0.239
Male	362 (51.9)	292 (48.6)	
Female	336 (48.1)	309 (51.4)	
Breastfeeding duration (months), *mean(SD)*	4.19±4.4	3.08±3.6	<0.001
Birth weight (kg), *mean(SD)*	3.21±0.4	3.14±0.5	0.016
Gestational age (weeks), *mean(SD)*	38.24±1.5	38.21±1.6	0.971

[a] Statistically significant differences ($p<0.05$), based on Mann-Whitney U test for two independent samples and Pearson's $\chi 2$ test for independence.
