# Supplementary material for: Metabolic Profile in Early Pregnancy Is Associated with Offspring Adiposity at 4 Years of Age: The Rhea Pregnancy Cohort Crete, Greece
Source: PLoS One. 2015 May 13;10(5):e0126327. doi: 10.1371/journal.pone.0126327 (PMC4430416; doi:10.1371/journal.pone.0126327)
Supplement: S2 Table — a Statistically significant differences (p<0.05), based on Mann-Whitney U test for two independent samples and Pearson's χ2 test for independence. (PDF) [file pone.0126327.s002.pdf]

S2 Table. Maternal and child characteristics of women who provided fasting blood samples in early pregnancy and those who did not in the Rhea pregnancy cohort Crete, Greece.

	**Fasting samples (n=348)**	**Non fasting samples (n=145)**	
**Maternal characteristics**			***P*- value**[a]
Maternal age (years), mean (SD)	29.90 (4.7)	30.50 (4.7)	0.160
Maternal education, *n(%)*			0.552
Low	58 (16.7)	23 (15.9)	
Medium	178 (51.1)	68 (46.9)	
High	112 (32.2)	54 (37.2)	
Mother's origin, *n (%)*			0.193
Greek	328 (94.2)	132 (91.0)	
Non greek	20 (5.8)	13 (9.0)	
Smoking status, *n (%)*			0.178
Smoker	101 (29.0)	51 (35.2)	
Non-smoker	24 7(71.0)	94 (64.8)	
Parity, *n (%)*			0.619
Primiparous	138 (39.7)	61 (42.1)	
Multiparous	210 (60.3)	84 (57.9)	
Pre-pregnancy BMI (kg/m^2), mean (SD)	24.62 (4.8)	24.67 (4.8)	0.957
Pre-pregnancy BMI categories			0.768
< 25 kg/m^2	228 (65.5)	97 (66.9)	
≥ 25 kg/m^2	120 (34.5)	48 (33.1)	
Gestational weight gain (kg), *n (%)*			0.204
Inadequate	67 (19.3)	34 (23.4)	
Adequete	134 (38.5)	62 (42.8)	
Excessive	147 (42.2)	49 (33.8)	
Delivery type, *n (%)*			0.564
Vaginal	166 (47.8)	73 (50.7)	
Caesarean	181 (52.2)	71 (49.3)	
**Child characteristics**			
Child gender, *n (%)*			0.087
Male	195 (56.0)	69 (47.6)	
Female	153 (44.0)	76 (52.4)	
Breastfeeding duration (months), mean(SD)	4.26 (4.3)	4.67 (4.6)	0.586
Birth weight (kg), mean (SD)	3.20 (0.4)	3.24 (0.4)	0.406
Gestational age (weeks), mean (SD)	38.25 (1.5)	38.39 (1.5)	0437

[a] Statistically significant differences ($p<0.05$), based on Mann-Whitney U test for two independent samples and Pearson's $\chi 2$ test for independence.
