# Supplementary material for: Metabolic Profile in Early Pregnancy Is Associated with Offspring Adiposity at 4 Years of Age: The Rhea Pregnancy Cohort Crete, Greece
Source: PLoS One. 2015 May 13;10(5):e0126327. doi: 10.1371/journal.pone.0126327 (PMC4430416; doi:10.1371/journal.pone.0126327)
Supplement: S3 Table — BMI, Body Mass Index; WC, Waist Circumference; TC, Total Cholesterol; LDL-C, Low Density Lipoprotein Cholesterol; HDL-C, High Density Lipoprotein Cholesterol; SBP, Systolic Blood Pressure; DBP, Diastolic Blood Pressure. All models were adjusted for child sex (except models using offspring systolic and diastolic blood pressure percentiles as an outcome) maternal age, education level, parity, smoking during pregnancy, gestational weight gain, birth weight, breastfeeding duration, TV watching at 4 years of age (hours/day) and paternal BMI. Models using offspring WC and sum of skinfolds as an outcome variable were also adjusted for child height, while those using offspring non-fasting lipid levels as an outcome were also adjusted for child BMI. Bold indicated statistically significant differences at p<0.05. (PDF) [file pone.0126327.s003.pdf]

S3 Table. Association of maternal pre-pregnancy obesity status with offspring cardiometabolic traits at 4 years of age after adjustment for paternal BMI in the Rhea pregnancy cohort Crete, Greece.

Offspring cardiometabolic traits at 4 years of age	**n**	**Pre-pregnancy overweight/obese (≥25 kg/m^2) (n=206)**
Adiposity outcomes		
		RR (95%CI)
Overweight/obese	133	**1.31 (0.94, 1.81)**
WC (cm) ≥ 90th pct	71	1.40 (0.90, 2.18)
		β-coeff. (95%CI)
Child BMI	609	**0.58 (0.24, 0.93)**
WC (cm)	601	**1.14 (0.34, 1.93)**
Sum of 4 Skinfolds (mm)	592	**4.34 (1.71, 6.97)**
Non-fasting lipid levels		***β-coeff. (95%CI)***
TC (mg/dl)	518	2.37 (-2.87, 7.62)
HDL-C (mg/dl)	518	0.82 (-1.31, 2.96)
Blood pressure levels		***β-coeff. (95%CI)***
SBP percentiles	482	0.12 (-0.33, 0.58)
DBP percentiles	482	-0.12 (-0.56, 0.31)

BMI, Body Mass Index; WC, Waist Circumference; TC, Total Cholesterol; LDL-C, Low Density Lipoprotein Cholesterol; HDL-C, High Density Lipoprotein Cholesterol; SBP, Systolic Blood Pressure; DBP, Diastolic Blood Pressucte; pct, percentile;

All models were adjusted for child sex (except models using offspring systolic and diastolic blood pressure percentiles as an outcome) maternal age, education level, parity, smoking during pregnancy, gestational weight gain, birth weight, breastfeeding duration TV watching at 4 years of age (hours/day) and paternal BMI. Models using offspring WC and sum of skinfolds as an outcome variable were also adjusted for child height, while those using offspring non-fasting lipid levels as an outcome were also adjusted for child BMI.

Bold indicated statistically significant differences at $p<0.05$.
