# Supplementary material for: Metabolic Profile in Early Pregnancy Is Associated with Offspring Adiposity at 4 Years of Age: The Rhea Pregnancy Cohort Crete, Greece
Source: PLoS One. 2015 May 13;10(5):e0126327. doi: 10.1371/journal.pone.0126327 (PMC4430416; doi:10.1371/journal.pone.0126327)
Supplement: S4 Table — BMI, Body Mass Index; WC, Waist Circumference; TC, Total Cholesterol; LDL-C, Low Density Lipoprotein Cholesterol; HDL-C, High Density Lipoprotein Cholesterol; SBP, Systolic Blood Pressure; DBP, Diastolic Blood Pressure; Model 1: adjusted for child sex. (except models using offspring systolic and diastolic blood pressure percentiles as an outcome). Model 2: model 1 further adjusted for maternal age, education level, parity and smoking during pregnancy Model 3: model 2 additionally adjusted for gestational weight gain, birth weight, breastfeeding duration, and TV watching at 4 years of age (hours/day). Models using offspring WC and sum of skinfolds as an outcome variable were also adjusted for child height, while those using offspring non-fasting lipid levels as an outcome were also adjusted for child BMI. Bold indicated statistically significant differences at p<0.05. (PDF) [file pone.0126327.s004.pdf]

S4 Table. Association of maternal pre-pregnancy obesity status with offspring cardiometabolic traits at 4 years of age, after excluding women with gestational diabetes (n=50), Rhea pregnancy cohort Crete, Greece.

		**Pre-pregnancy overweight/obese (≥25 kg/m^2) (n=209)**		
Offspring cardiometabolic traits at 4 years of age	**n**	**Model 1**	**Model 2**	**Model 3**
Adiposity outcomes				
		RR (95%CI)	***RR (95%CI)***	***RR (95%CI)***
Overweight/obese	122	**1..65 (1.21, 2.26)**	**1.57 (1.14, 2.18)**	1.39 (0.98, 1.97)
WC (cm) ≥ 90th pct	63	**2.07 (1.32, 3.25)**	**1.90 (1.19, 3.04)**	1.51 (0.92, 2.46)
		β-coeff. (95%CI)	***β-coeff. (95%CI)***	***β-coeff. (95%CI)***
Child BMI	568	**0.81 (0.44, 1.17)**	**0.78 (0.42, 1.14)**	**0.71 (0.32, 1.10)**
WC (cm)	556	**1.77 (0.84, 2.70)**	**1.76 (0.84, 2.68)**	**1.35 (0.45, 2.25)**
Sum of 4 Skinfolds (mm)	551	**5.52 (2.85, 8.19)**	**5.05 (2.34, 7.78)**	**4.38 (1.42, 7.35)**
Non-fasting lipid levels		***β-coeff. (95%CI)***	***β-coeff. (95%CI)***	***β-coeff. (95%CI)***
TC(mg/dl)	475	2.73 (-2.28, 7.74)	2.74 (-2.47, 7.96)	1.84 (-3.87, 7.55)
HDL-C(mg/dl)	475	0.43 (-1.64, 2.50)	0.39 (-1.78, 2.55)	0.22 (-2.13, 2.57)
Blood pressure levels		***β-coeff. (95%CI)***	***β-coeff. (95%CI)***	***β-coeff. (95%CI)***
SBP percentiles	438	0.35 (-0.11, 0.81)	0.40 (-0.07, 0.88)	0.32 (-0.16, 0.81)
DBP percentiles	438	-0.02 (-0.46, 0.42)	-0.07 (-0.51, 0.40)	-0.05 (-0.50, 0.40)

BMI, Body Mass Index; WC, Waist Circumference; TC, Total Cholesterol; LDL-C, Low Density Lipoprotein Cholesterol; HDL-C, High Density Lipoprotein Cholesterol; SBP, Systolic Blood Pressure; DBP, Diastolic Blood Pressure; pct, percentile;

Model 1: adjusted for child sex.(except models using offspring systolic and diastolic blood pressure percentiles as an outcome)

Model 2: model 1 further adjusted for maternal age, education level, parity and smoking during pregnancy

Model 3: model 2 additionally adjusted for gestational weight gain, birth weight, breastfeeding duration, and TV watching at 4 years of age (hours/day). Models using offspring WC and sum of skinfolds as an outcome variable were also adjusted for child height, while those using offspring non-fasting lipid levels as an outcome were also adjusted for child BMI. Bold indicated statistically significant differences at $p<0.05$.
