# Supplementary material for: Metabolic Profile in Early Pregnancy Is Associated with Offspring Adiposity at 4 Years of Age: The Rhea Pregnancy Cohort Crete, Greece
Source: PLoS One. 2015 May 13;10(5):e0126327. doi: 10.1371/journal.pone.0126327 (PMC4430416; doi:10.1371/journal.pone.0126327)
Supplement: S5 Table — BMI, Body Mass Index; WC, Waist Circumference; TC, Total Cholesterol; LDL-C, Low Density Lipoprotein Cholesterol; HDL-C, High Density Lipoprotein Cholesterol; SBP, Systolic Blood Pressure; DBP, Diastolic Blood Pressure; Model 1: adjusted for child sex. (except models using offspring systolic and diastolic blood pressure percentiles as an outcome) Model 2: model 1 further adjusted for maternal age, education level, parity, smoking during pregnancy and pre-pregnancy BMI. Model 3: model 2 additionally adjusted for gestational weight gain, birth weight, breastfeeding duration, and TV watching at 4 years of age (hours/day). Models using offspring WC and sum of skinfolds as an outcome variable were also adjusted for child height, while those using offspring non-fasting lipid levels as an outcome were also adjusted for child BMI. Bold indicated statistically significant differences at p<0.05 (PDF) [file pone.0126327.s005.pdf]

S5 Table. Association of maternal fasting lipid profile in early pregnancy with offspring cardiometabolic traits at 4 years of age, after excluding women with gestational diabetes (n=25), Rhea pregnancy cohort Crete, Greece.

		**Fasting TC levels in early pregnancy (per increase in 40 mg/dL) (n=323)**			**Fasting LDL-C levels in early pregnancy (per increase in 15 mg/dL) (n=323)**		
Offspring cardiometabolic traits at 4 years of age	**n**	**Model 1**	**Model 2**	**Model 3**	**Model 1**	**Model 2**	**Model 3**
Adiposity outcomes							
		RR (95%CI)	***RR (95%CI)***	***RR (95%CI)***	***RR (95%CI)***	***RR (95%CI)***	***RR (95%CI)***
Overweight/obese	59	1.18 (0.92, 1.53)	1.23 (0.95, 1.61)	1.37 (0.99, 1.89)	1.05 (0.93, 1.19)	1.06 (0.93, 1.21)	1.08 (0.92, 1.27)
WC (cm) ≥ 90th pct	27	1.05 (0.71, 1.56)	1.04 (0.70, 1.57)	1.24 (0.72, 2.11)	1.04 (0.86, 1.27)	1.06 (0.86, 1.30)	1.11 (0.87, 1.41)
		β-coeff. (95%CI)	***β-coeff. (95%CI)***	***β-coeff. (95%CI)***	***β-coeff. (95%CI)***	***β-coeff. (95%CI)***	***β-coeff. (95%CI)***
Child BMI	323	0.06 (-0.16, 0.29)	0.08 (-0.16, 0.32)	0.03 (-0.22, 0.28)	0.01 (-0.10, 0.13)	0.02 (-0.11, 0.16)	-0.02 (-0.17, 0.12)
WC (cm)	323	0.29 (-0.37, 0.95)	0.30 (-0.42, 1.01)	0.42 (-0.26, 1.11)	0.10 (-0.25, 0.44)	0.11 (-0.27, 0.50)	0.14 (-0.25, 0.53)
Sum of 4 Skinfolds (mm)	316	2.52 (0.78, 4.26)	2.82 (0.98, 4.65)	**3.19 (1.22, 5.15)**	0.78 (-0.15, 1.71)	0.85 (-0.15, 1.86)	1.05 (-0.02, 2.13)
Non-fasting lipid levels		***β-coeff. (95%CI)***	***β-coeff. (95%CI)***	***β-coeff. (95%CI)***	***β-coeff. (95%CI)***	***β-coeff. (95%CI)***	***β-coeff. (95%CI)***
TC(mg/dl)	269	**3.45 (0.10, 6.81)**	3.44 (-0.12, 7.00)	3.68 (-0.38, 7.75)	2.28 (0.60, 3.96)	2.20 (0.44, 3.95)	**2.34 (0.22, 4.47)**
HDL-C(mg/dl)	269	-0.47 (-1.89, 0.94)	-0.83 (-2.32, 0.67)	-1.15 (-2.93, 0.62)	-0.43 (-1.10, 0.24)	-0.68 (-1.28, 0.12)	-0.70 (-1.56, 0.14)
Blood pressure levels		***β-coeff. (95%CI)***	***β-coeff. (95%CI)***	***β-coeff. (95%CI)***	***β-coeff. (95%CI)***	***β-coeff. (95%CI)***	***β-coeff. (95%CI)***
SBP percentiles	259	-0.08 (-0.42, 0.25)	-0.12 (-0.47, 0.23)	-0.14 (-0.52, 0.24)	-0.06 (-0.22, 0.10)	-0.08 (-0.25, 0.09)	-0.09 (-0.27, 0.09)
DBP percentiles	259	-0.12 (-0.32, 0.08)	-0.16 (-0.36, 0.05)	-0.16 (-0.39, 0.05)	-0.04 (-0.14, 0.05)	-0.07 (-0.17, 0.03)	-0.07 (-0.18, 0.03)

BMI, Body Mass Index; WC, Waist Circumference; TC, Total Cholesterol; LDL-C, Low Density Lipoprotein Cholesterol; HDL-C, High Density Lipoprotein Cholesterol; SBP, Systolic Blood Pressure; DBP, Diastolic Blood Pressure; pct, percentile;

Model 1: adjusted for child sex.(except models using offspring systolic and diastolic blood pressure percentiles as an outcome)

Model 2: model 1 further adjusted for maternal age, education level, parity, smoking during pregnancy and pre-pregnancy BMI

Model 3: model 2 additionally adjusted for gestational weight gain, birth weight, breastfeeding duration, and TV watching at 4 years of age (hours/day). Models using offspring WC and sum of skinfolds as an outcome variable were also adjusted for child height, while those using offspring non-fasting lipid levels as an outcome were also adjusted for child BMI. Bold indicated statistically significant differences at $p<0.05$
