# Supplementary material for: Metabolic Profile in Early Pregnancy Is Associated with Offspring Adiposity at 4 Years of Age: The Rhea Pregnancy Cohort Crete, Greece
Source: PLoS One. 2015 May 13;10(5):e0126327. doi: 10.1371/journal.pone.0126327 (PMC4430416; doi:10.1371/journal.pone.0126327)
Supplement: S6 Table — BMI, Body Mass Index; WC, Waist Circumference; TC, Total Cholesterol; LDL-C, Low Density Lipoprotein Cholesterol; HDL-C, High Density Lipoprotein Cholesterol; SBP, Systolic Blood Pressure; DBP, Diastolic Blood Pressure;. Model 1: adjusted for child sex (except models using offspring systolic and diastolic blood pressure percentiles as an outcome). Model 2: model 1 further adjusted for maternal age, education level, parity, smoking during pregnancy and pre-pregnancy BMI. Model 3: model 2 additionally adjusted for gestational weight gain, birth weight, breastfeeding duration, and TV watching at 4 years of age (hours/day). Models using offspring WC and sum of skinfolds as an outcome variable were also adjusted for child height, while those using offspring non-fasting lipid levels as an outcome were also adjusted for child BMI. Bold indicated statistically significant differences p<0.05. (PDF) [file pone.0126327.s006.pdf]

S6 Table. Association of maternal blood pressure levels in early pregnancy with offspring cardiometabolic traits at 4 years of age, after excluding women with gestational diabetes (n=48), Rhea pregnancy cohort Crete, Greece

		**SBP in early pregnancy (per increase in 10 mm Hg) (n=488)**			**DBP in early pregnancy (per increase in 10 mm Hg) (n=488)**		
Offspring cardiometabolic traits at 4 years of age	**n**	**Model 1**	**Model 2**	**Model 3**	**Model 1**	**Model 2**	**Model 3**
Adiposity outcomes							
		RR (95%CI)	***RR (95%CI)***	***RR (95%CI)***	***RR (95%CI)***	***RR (95%CI)***	***RR (95%CI)***
Overweight/obese	103	**1.23 (1.04, 1.44)**	**1.22 (1.04, 1.44)**	**1.22 (1.02, 1.45)**	**1.25 (1.06, 1.47)**	**1.23 (1.04, 1.46)**	**1.23 (1.03, 1.47)**
WC (cm) ≥ 90th pct	54	1.22 (0.97, 1.54)	1.18 (0.93, 1.51)	1.07 (0.84, 1.36)	1.22 (0.96, 1.56)	1.18 (0.92, 1.53)	1.14 (0.88, 1.48)
		β-coeff. (95%CI)	***β-coeff. (95%CI)***	***β-coeff. (95%CI)***	***β-coeff. (95%CI)***	***β-coeff. (95%CI)***	***β-coeff. (95%CI)***
Child BMI	462	**0.18 (0.00, 0.36)**	**0.18 (0.00, 0.35)**	0.13 (-0.05, 0.32)	0.15 (-0.03, 0.33)	0.15 (-0.02, 0.32)	0.12 (-0.04, 0.30)
WC (cm)	458	**0.48 (0.05, 0.92)**	**0.48 (0.05, 0.92)**	0.27 (-0.13, 0.68)	0.44 (-0.02, 0.91)	0.40 (-0.05, 0.86)	0.32 (-0.05, 0.71)
Sum of 4 Skinfolds (mm)	451	**1.85 (0.40, 3.30)**	**1.74 (0.31, 3.18)**	1.43 (-0.09, 2.95)	**2.30 (1.02, 3.57)**	**2.16 (0.93, 3.39)**	**1.93 (0.71, 3.16)**
Non-fasting lipid levels		***β-coeff. (95%CI)***	***β-coeff. (95%CI)***	***β-coeff. (95%CI)***	***β-coeff. (95%CI)***	***β-coeff. (95%CI)***	***β-coeff. (95%CI)***
TC(mg/dl)	395	-0.70 (-3.07, 1.67)	-0.67 (-3.07, 1.72)	-1.29 (-3.76, 1.17)	-1.62 (4.50, 1.25)	-1.36 (-4.20, 1.47)	-1.79 (-4.77, 1.19)
HDL-C(mg/dl)	395	0.51 (-0.46, 1.48)	0.54 (-0.43, 1.51)	0.72 (-0.34, 1.79)	-0.49 (-1.60, 0.62)	-0.24 (-1.37, 0.88)	-0.22 (-1.40, 0.95)
Blood pressure levels		***β-coeff. (95%CI)***	***β-coeff. (95%CI)***	***β-coeff. (95%CI)***	***β-coeff. (95%CI)***	***β-coeff. (95%CI)***	***β-coeff. (95%CI)***
SBP percentiles	360	**0.18 (0.00, 0.37)**	**0.19 (0.00, 0.38)**	**0.23 (0.02, 0.43)**	0.20 (-0.01, 0.41)	0.17 (-0.04, 0.39)	0.17 (-0.04, 0.39)
DBP percentiles	360	0.07 (-0.04, 0.19)	0.07 (-0.04, 0.19)	0.09 (-0.02, 0.21)	0.10 (-0.03, 0.23)	0.08 (-0.05, 0.21)	0.08 (-0.05, 0.21)

BMI, Body Mass Index; WC, Waist Circumference; TC, Total Cholesterol; LDL-C, Low Density Lipoprotein Cholesterol; HDL-C, High Density Lipoprotein Cholesterol;; SBP, Systolic Blood Pressure; DBP, Diastolic Blood Pressure; pct, percentile;

Model 1: adjusted for child sex.(except models using offspring systolic and diastolic blood pressure percentiles as an outcome)

Model 2: model 1 further adjusted for maternal age, education level, parity, smoking during pregnancy and pre-pregnancy BMI

Model 3: model 2 additionally adjusted for gestational weight gain, birth weight, breastfeeding duration, and TV watching at 4 years of age (hours/day). Models using offspring WC and sum of skinfolds as an outcome variable were also adjusted for child height, while those using offspring non-fasting lipid levels as an outcome were also adjusted for child BMI. Bold indicated statistically significant differences at $p<0.05$
